# The DNA G-Quadruplex-Stabilizing Ligand TMPyP4 Inhibits Maize Radicle Growth by Modulating Reactive Oxygen Species Homeostasis

**DOI:** 10.3390/life16060910

**Published:** 2026-05-28

**Authors:** Chenxi Meng, Meng Sun, Ming Wang, Haiyan Zhang, De Xue, Jinjie Guo, Jingtang Chen, Fuchao Jiao

**Affiliations:** 1 College of Agronomy, Qingdao Agricultural University, Qingdao 266109, China; 2 Office of Academic Discipline Development, Shandong Agriculture and Engineering University, Zibo 255300, China; 3 Shandong Key Laboratory of Maize Biological Breeding, Qingdao 266109, China; 4 The Characteristic Laboratory of Crop Germplasm Innovation and Application, Provincial Department of Education, Qingdao 266109, China; 5 Zibo Boxin Agricultural Technology Co., Ltd., Zibo 256401, China; 6 State Key Laboratory of North China Crop Improvement and Regulation, Hebei Sub-Center for National Maize Improvement Center, College of Agronomy, Hebei Agricultural University, Baoding 071001, China

**Keywords:** TMPyP4, maize, ROS, BG4-ChIP-seq, DG4

## Abstract

G-quadruplex (DG4) is folded in guanine-rich DNA sequences and regulates DNA replication and transcription. Although bioinformatics analyses have predicted the presence of DG4 in maize, its biological functions remain largely unexplored. In this study, we treated maize seedlings with 0, 100, 200, and 300 μM TMPyP4, a DG4-stabilizing ligand, and observed that TMPyP4 inhibits radicle growth by increasing reactive oxygen species (ROS) levels and inducing DNA fragmentation in radicle cells. Transcriptomic RNA-seq revealed that TMPyP4 modulated the expression of 1614 genes in maize radicle cells, which were predominantly associated with redox reactions, membrane components, and secondary metabolic pathways. BG4-ChIP-seq analysis demonstrated that DG4 structures are evenly distributed across the ten chromosomes of the maize genome, occupying 22,449 loci and showing significant enrichment for specific DG4-binding motifs. Integrative analysis of RNA-seq data and BG4-ChIP-seq identified 944 differentially expressed genes, which were significantly enriched in pathways related to redox reactions and secondary metabolism. Collectively, these findings suggest that DG4-stabilizing ligands regulate maize radicle growth by modulating ROS homeostasis, providing critical insights into the functional roles of DG4s in maize.

## 1. Introduction

DNA guanine quadruplex (DG4) is one kind of secondary structure that frequently forms in guanine-rich DNA sequences. The function of DG4 has been extensively studied in cancer cells but has only recently been predicted in plant genomes. Putative quadruplex-forming G-rich sequences have been identified in arabidopsis, rice [1], wheat [2], and barley [3]; abundant putative DG4s are enriched within 1 kb upstream of the start codon, indicating a potential regulatory role in gene expression. In maize, over 43,000 putative DG4s have been identified in both telomeric repeat DNA sequences and non-telomeric genomic loci, such as the 5′ UTR and the first intron on the antisense strand. Maize putative DG4s are present in genes that are related to energy status, oxidative stress and hypoxia [4], suggesting a common capacity of DG4s in regulating maize development. However, the function of these putative DG4s has not been clearly elucidated.

DG4 regulates both DNA replication and transcription. The folding of DG4 is influenced by the cellular environment and binding proteins; helicases, such as Pif1 [5], BLM [6], and FANCJ [7], are required to resolve DG4. For example, in yeast, the lack of Pif1 to unwind DG4 results in these higher-order structures physically impeding replication fork progression, leading to DNA double-strand breaks [8]. In addition to stalling DNA replication, DG4 can both impede transcription [9] and promote transcription [10]. When located on the *c-MYC* template strand, DG4 represses transcription by blocking the movement of RNA polymerase, while when located on the *c-MYC* non-template strand, DG4 enhances R-loop formation and promotes transcription [8,11].

To understand the formation characteristics and biological functions of DG4, ligands with high G4-structure-specific binding affinity have been developed. Cancer cellular growth is influenced when treated with these DG4 ligands. Pyridostatin (PDS) inhibits cancer cellular growth by causing DNA damage at G4 sites in a replication- and transcription-dependent manner [12]. N-methyl-4-pyridyl (TMPyp4) stacks with the external G-quartet and stabilizes both parallel and antiparallel DG4 conformations [13]. TMPyP4 effectively inhibits the expression of *c-MYC* in multiple cancer cells, impeding cancer cell growth [14]. TMPyP4 inhibits colorectal cancer through cell cycle arrest by targeting DG4 [15]. APTO-253 has the same function as TMPyP4 [16].

ChIP-seq with the BG4 antibody is widely used in DG4 detection. BG4 only binds to DNA sequences rich in guanine (G) that can form G4 structures, but it does not bind to their complementary cytosine (C)-rich sequences or random sequences, serving as a key tool for analyzing the distribution, biological functions, and molecular regulatory mechanisms of DG4s [17,18]. BG4 has been applied in the localization of G4 structures in human and rice DNA [19,20]. In spontaneously immortalized HaCaT keratinocytes and human cervical carcinoma cells, about 10,000 and 8000 DG4s were identified, respectively. The majority of DG4s were found in nucleosome-depleted chromatin regions and were enriched in regulatory regions, particularly within promoter regions and around transcription start sites [21]. In rice, 23,685 DG4 peaks were identified and showed a genic-dependent enrichment of epigenomic features. DG4s show differential DNA methylation between transposable elements (TEs) and non-TE genes. DG4s in promoter regions are positively correlated with gene expression, whereas DG4s in gene bodies are negatively correlated with gene expression [22].

Regions containing DG4s are highly sensitive to ROS-induced damage. Due to its low oxidation potential and strong binding affinity with transition metal ions, guanine is particularly susceptible to ROS attack. When guanine is oxidized by ROS, it forms 8-oxoguanine (OG), which represses the stability of DG4s [23]. However, other research shows that OG may stabilize the formation of DG4s [24]. For example, oxidative stress modulates DG4 stability and structure in the human *BCL2* promoter [25]. Oxidative damage triggers the formation of R-loop and G4 structures in a BLM helicase-dependent manner [26]. H_2_O_2_ can enhance TMPyP4-induced DNA damage and provoke stronger DNA damage response in cancer cells [27]. TMPyP4 induces DNA damage and provokes stronger DDR in cancer cells but not in normal cells [28]. In ovarian cancer cells, TMPyP4-induced ROS elevation exacerbates mitochondrial dysfunction and DNA damage, ultimately inhibiting proliferation and inducing apoptosis [29]. In maize, the homeostasis of ROS plays a finely tuned and complex regulatory role in seed germination and seedling growth. ZmbZIP89 promotes the expression of *ZmPRX47*, increasing lateral root length and enhancing drought resistance by regulating the production of root ROS homeostasis [30]. Under drought and salt stress, overexpression of *ZmSRG7* improves root growth by reducing H_2_O_2_, MDA, and O_2_^−^ levels and by increasing SOD and POD activities [31]. In this study, we hypothesized that the DG4-stabilizing ligand TMPyP4 influences maize radicle growth by regulating DG4 formation and ROS levels. We performed a series of physical and molecular analyses, including RNA-seq and BG4 Chip-seq, to provide a reference for elucidating the biological functions of DG4s.

## 2. Materials and Methods

### 2.1. Measurement of Radicle Length

Maize B73 is an important reference inbred line in maize genetics and breeding research. B73 seeds were collected from our laboratory. B73 seeds were first sterilized with sodium hypochlorite, then rinsed with distilled water, and soaked for 24 h. Twenty seeds were placed in each germination box, and 20 mL of the corresponding treatment solution was added. Four treatment groups were established: H_2_O (control, CK) and 100, 200, and 300 μM TMPyP4 (Y35384, Shanghai yuanye Bio-Technology Co., Ltd., Shanghai, China). The seeds were cultured in a light incubator at 25 °C under a 12 h light/12 h dark photoperiod for 7 days, after which radicle length was measured.

### 2.2. Determination of ROS Content in Maize Radicles

Radicles (0.1 g each) from the control and 300 μM TMPyP4 treatment groups were rinsed with PBS and homogenized. After equilibrating a plant reactive oxygen species (ROS) ELISA kit plate (MK30062A, Jiangsu Sumeike Biotechnology Co., Ltd., Nanjing, China.) to room temperature, standard, sample, and blank wells were set up. First, 50 mL of each sample was added, followed by HRP-labeled detection antibody, and the plate was incubated at 37 °C for 60 min. The plate was washed five times, and the substrate was added for incubation in the dark. After the stop solution was added, the optical density (OD) value was measured at 450 nm using a microplate reader within 15 min. A regression curve was generated using standard concentrations and OD values to calculate ROS concentrations in the samples.

### 2.3. Single-Cell Gel Electrophoresis

Maize radicles treated with TMPyP4 were collected, and nuclei were mechanically isolated on ice to prepare a suspension. A first layer of 0.6% normal-melting-point agarose was prepared. The nuclear suspension was mixed with 0.7% low-melting-point agarose, spread as a second layer, and allowed to solidify. The slides were immersed in pre-chilled lysis buffer and incubated at 4 °C in the dark for 2 h, followed by unwinding in electrophoresis buffer for 30 min. Electrophoresis was performed at 25 V in an ice bath for 20–30 min. The slides were rinsed with Tris-HCl buffer three times, dehydrated in absolute ethanol, and stained with ethidium bromide for 20 min. Observations were made under a fluorescence microscope at 510–560 nm wavelength, and the DNA migration length was measured.

### 2.4. Transcriptome Sequencing of Maize Radicles Treated with TMPyP4

Radicles from B73 seeds treated with 0, 100, and 300 μM TMPyP4 were collected; each group had three repetitions. RNA was extracted, reverse transcribed into cDNA, and amplified. Adaptor fragments were added to construct libraries, which were sequenced on the Illumina Novaseq™ 6000 platform. Raw data were filtered, which were then aligned to the B73 reference genome. Gene expression levels were quantified as FPKM. Differentially expressed genes were identified based on |log2FC| ≥ 1 and q < 0.05, followed by GO functional enrichment and KEGG pathway enrichment analyses. Sequencing service outsourcing was conducted by LC-Bio Technologies (Hangzhou) Co., Ltd., Hangzhou, China.

### 2.5. Validation by Quantitative Real-Time PCR

Primers were designed using Primer 5 and validated for specificity (Table S1). GADPH was used as the internal control, with three biological and three technical replicates. RNA was extracted (AG21019, Accurate Biotechnology (Hunan) Co., Ltd., Changsha, China) and reverse transcribed into cDNA (AG11728, Accurate Biotechnology (Hunan) Co., Ltd., Changsha, China) using a specific system. qPCR reaction mixtures (AG11702, Accurate Biotechnology (Hunan) Co., Ltd., Changsha, China) were prepared and amplified on a real-time PCR system using a specified protocol. Primer specificity was verified by amplification and melting curves, and relative gene expression levels were calculated based on CT values to validate the reliability of RNA-seq results.

### 2.6. BG4-ChIP-Seq Analysis

The BG4 antibody (MABE917, Merck KgaA, Sigma-Aldrich, Saint Louis, MO, USA) was used for BG4-ChIP-seq analysis. B73 seeds were sown in a 3:1 nutrient soil–vermiculite mixture and grown in darkness at 25 °C with 70% humidity for 7 days. Leaves were collected, frozen in liquid nitrogen, and stored at −80 °C. Two grams of each sample was fixed with formaldehyde, and crosslinking was terminated with glycine. After grinding, the samples were treated with cell lysis buffer to obtain nuclear suspensions. Chromatin DNA was fragmented by sonication, and immunoprecipitation was performed for the Input and IP groups. Sequencing libraries were constructed and subjected to quality control, followed by sequencing on the MGISEQ-T7 platform. Raw sequencing data were processed for quality control using fastp and aligned to the genome with RSeQC. Peak calling, GO/KEGG enrichment analysis, and motif scanning were performed using Macs2 (Version 2.1.1), Bedtools (Version 2.25.0), and other software. Sequencing service outsourcing was performed by Wuhan Kangce Technology Co., Ltd., Wuhan, China.

## 3. Results

### 3.1. Maize Seedling Growth Is Affected by TMPyP4

**Figure 1:**
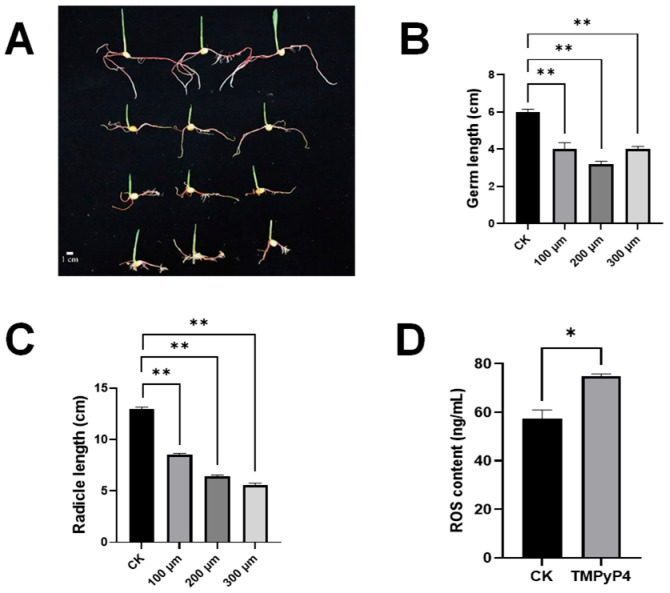
Phenotypic and ROS content analysis of B73 following treatment with varying concentrations of TMPyP4. (**A**) Photos of seedlings treated with TMPyP4. TMPyP4 concentrations from top to bottom were 0 μM, 100 μM, 200 μM, and 300 μM. Scale bar: 1 cm. (**B**) Data analysis of coleoptile length (*N* ≥ 10, ** *p* < 0.01). (**C**) Data analysis of radicle length (*N* ≥ 10, ** *p* < 0.01). (**D**) Data analysis of ROS content in primary roots treated with 300 μM TMPyP4 (*N* ≥ 10, * *p* < 0.05).

To investigate the function of maize DG4, maize seedlings were treated with the DG4 stabilizer TMPyP4 at concentrations of 0 μM (control), 100 μM, 200 μM, and 300 μM (Figure 1A). The germ length in the control was about 6.0 cm, while it was about 4.0 cm, 3.0 cm, and 3.8 cm after the 100 μM, 200 μM, and 300 μM TMPyP4 treatments, respectively (Figure 1B). The radicle length in the control was about 13 cm, while it was about 8 cm, 6 cm and 5 cm after the 100 μM, 200 μM, and 300 μM TMPyP4 treatments, respectively (Figure 1C). The length of the germ shoot decreased by 37.6%, 47.8 and 35.7%, respectively, while the length of the germ root decreased by 45.1%, 57.3% and 66.2%, respectively. These results indicate that TMPyP4 reduced the lengths of both embryonic shoots and radicles concentration-dependently.

To explore whether TMPyP4 is associated with reactive oxygen species (ROS), the ROS content in the radicles treated with 300 μM TMPyP4 was measured (Figure 1D). The ROS content in the control group was approximately 57.5 ng/mL, whereas that in the treated radicles was approximately 74.6 ng/mL, representing a 22.9% increase in the treatment group. These results indicate that TMPyP4 elevates ROS levels in maize radicle somatic cells and may disrupt redox homeostasis.

**Figure 2:**
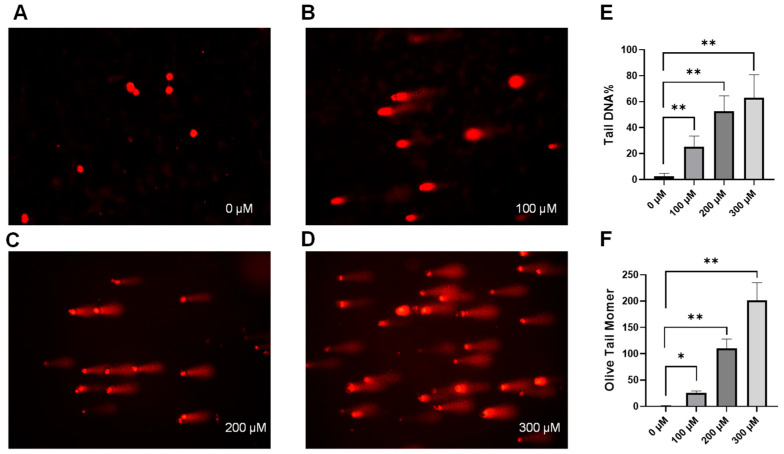
Single-cell gel electrophoresis and data analysis of B73 radicle somatic cells treated with different concentrations of TMPyP4. (**A**–**D**) Single-cell gel electrophoresis of B73 radicle somatic cells treated with 0 μM TMPyP4, 100 μM TMPyP4, 200 μM TMPyP4 and 300 μM TMPyP4, respectively. (**E**) Data analysis of tail DNA percentage. (**F**) Data analysis of Olive Tail Momer. (*N* ≥ 10, * *p* < 0.05, ** *p* < 0.01).

To investigate whether TMPyP4 induces DNA damage, single-cell gel electrophoresis was performed in radicle somatic cells. Radicle cells in the control exhibited no comet tails, indicating intact nuclear DNA (Figure 2A). In contrast, all treatment groups displayed typical comet morphologies; in addition, the comet rate, tail length, and fluorescence intensity significantly increased in a concentration-dependent manner (Figure 2B–D). Statistical analysis revealed that the percentage of tail DNA in all treatment groups was significantly higher than that in the control group (Figure 2E). Olive tail momer (OTM) in the treatment groups was higher than that in the control and increased significantly with rising TMPyP4 concentration (Figure 2F). The percentage of tail DNA and OTM data both indicate that TMPyP4 induced DNA damage.

### 3.2. Transcriptome Alterations Induced by TMPyP4 Treatment

**Figure 3:**
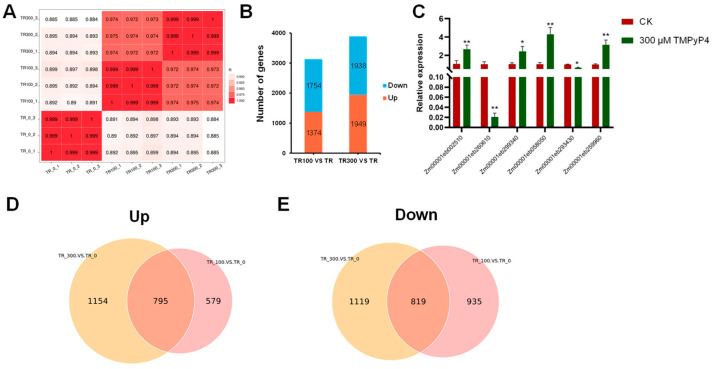
Transcriptome analysis of radicle samples. (**A**) Transcriptome PCA correlation coefficient plot, where both the x-axis and y-axis represent individual samples; proximity to red (correlation coefficient close to 1) indicates a higher correlation. (**B**) Total number of upregula ted and downregulated genes in different treatment groups. Orange represents upregulated genes, and blue represents downregulated genes. (**C**) qRT-PCR validation of transcriptome results. Red indicates the control group (CK, 0 μM treatment), and green indicates the 300 μM TMPyP4 treatment group. (**D**) Venn diagram of downregulated genes in the 100 μM treatment group (TR_100) and the 300 μM treatment group (TR_300). In the Venn diagram, the values in the overlapping regions represent commonly identified differentially expressed genes, while the values in the non-overlapping regions correspond to genes uniquely differentially expressed after each respective treatment. (**E**) Venn diagram of upregulated genes in the 100 μM treatment group (TR_100) and the 300 μM treatment group (TR_300). In the Venn diagram, the values in the overlapping regions represent commonly identified differentially expressed genes, while the values in the non-overlapping regions correspond to genes uniquely differentially expressed after each respective treatment. (* *p* < 0.05, ** *p* < 0.01).

To investigate which genes were influenced by TMPyP4, transcriptome analysis was performed on nine radicle samples, including a control group (TR_0), a 100 μM treatment group (TR_100), and a 300 μM treatment group (TR_300), each comprising three biological replicates. High correlation (Figure 3A) and PCA (Figure S1) were observed within the treatment groups, whereas significant differences were noted between the treatment groups and the control group. Compared with the control group, 1374 genes were upregulated and 1754 genes were downregulated in the 100 μM TMPyP4 treatment group, while 1949 genes were upregulated and 1938 genes were downregulated in the 300 μM TMPyP4 treatment group (Figure 3B). Venn analysis across treatment groups revealed 819 commonly downregulated genes (Figure 3D) and 795 commonly upregulated genes (Figure 3E).

GO enrichment analysis identified 322 significantly enriched terms. In terms of biological process (BP), the DEGs were primarily involved in responses to oxygen-containing compounds and oxidation–reduction processes. Regarding cellular component (CC), the DEGs were predominantly localized to the intrinsic component of the membrane, integral component of the membrane, and plasma membrane parts. For molecular function (MF), the DEGs were mainly enriched in oxidoreductase activity, transmembrane transporter activity, and monooxygenase activity. KEGG pathway analysis of the upregulated genes revealed 30 significantly enriched pathways, including phenylpropanoid biosynthesis, MAPK signaling pathway—plant, and glutathione metabolism. GO and KEGG analyses of the downregulated genes are presented in the appendix figure.

To validate the reliability of the transcriptome results, six genes—Zm00001eb002510, Zm00001eb260610, Zm00001eb299340, Zm00001eb058050, Zm00001eb293430, and Zm00001eb259960—were randomly selected for quantitative real-time PCR (qRT-PCR) validation. The qRT-PCR results were consistent with the transcriptome data (Figure 3C). These findings indicate that TMPyP4 significantly alters gene expression in maize seedlings. Specifically, the upregulated genes primarily exert effects by inducing redox reactions and modulating membrane component functions and secondary metabolic pathways, which may be associated with TMPyP4-mediated ROS accumulation.

### 3.3. BG4-ChIP-Seq Identified DG4 Motifs

**Figure 4:**
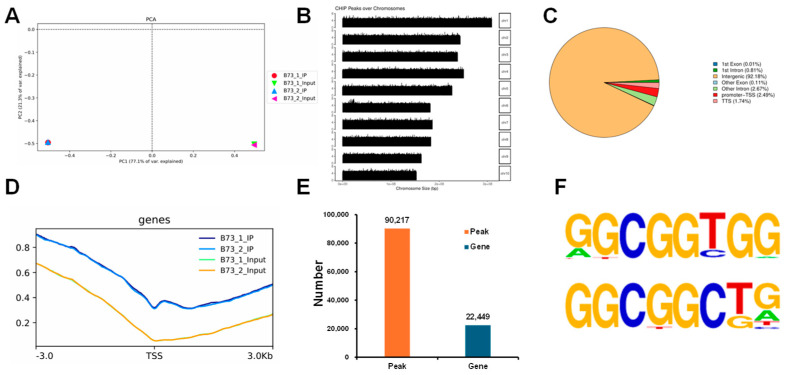
BG4-ChIP-seq analysis. (**A**) PCA plot of sequencing samples, demonstrating clear separation between the IP and Input groups, with tight clustering of replicate samples. (**B**) Distribution of signal peaks across the whole genome chromosomes. The x-axis represents the length of the chromosomes, the right side indicates the chromosome numbers, and the left y-axis represents the peak value on each chromosome. (**C**) Proportion of signal peaks in different genomic regions, with intergenic regions showing the highest proportion, and significant enrichment observed in promoter–TSS regions. (**D**) Line graph illustrating peak signal enrichment within ±3 kb of transcription start sites (TSSs), showing a sharp peak at the TSS in the IP group, exceeding that of the Input group. (**E**) Statistical summary of signal peaks, with orange representing identified peak sites and blue representing the number of associated genes. (**F**) Motif analysis of IP-G4 sites, identifying a typical G-rich sequence consistent with the core features of G4 structures.

To validate the presence of genome DG4 structures, BG4-ChIP-seq was performed in vitro. Clean reads were aligned to the B73 reference genome using STAR (Version 2.5.3a) software, with approximately 81.95% of reads from the IP sample and 81.5% from the Input sample successfully mapping to the reference genome (Table S2). Principal component analysis (PCA) revealed a clear separation between the IP and Input groups (Figure 4A).

A total of 90,217 high-confidence peaks were identified, corresponding to 22,449 associated genes (Figure 4E). Peak lengths ranged from 192 to 4166 bp and were distributed across all 10 chromosomes (Figure 4B). Genomic regional distribution analysis showed that the peaks were enriched in the promoter region (promoter–TSS), accounting for 2.49%, with lower proportions observed in the first intron, other introns, and the first exon regions (Figure 4C). This result suggests that the peaks are primarily involved in the regulation of gene transcription. Metaplot analysis of TSS enrichment revealed a sharp peak at the TSS in the IP group, which was higher than that in the Input group (Figure 4D), confirming that G4 structures are highly enriched around transcription start sites and further supporting their central role in transcriptional regulation.

Sequences within the peak intervals were extracted using HOMER (version 4.10), and G4-related motifs were significantly enriched (Figure 4F), indicating that BG4 specifically binds to G4 structures. The identified motifs were compared against known transcription factor databases, and a total of 137 transcription factors were found, including AT3G57600 (AP2/EREBP), ESE1 (AP2/EREBP), and MYB92 (MYB).

### 3.4. Integrative Analysis Identified Affected Genes Containing DG4

**Figure 5:**
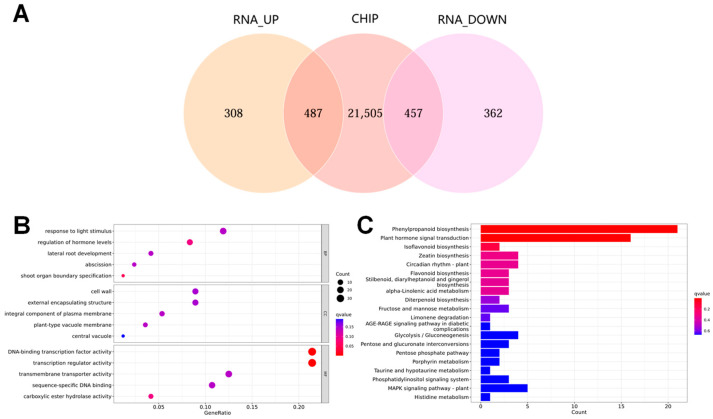
Integrated analysis of RNA-seq and ChIP-seq data. (**A**) Venn diagram of integrated genes. (**B**) GO analysis of downregulated genes. (**C**) KEGG analysis of downregulated genes.

To elucidate DEGs containing DG4, an integrative analysis of RNA-seq and ChIP-seq data was conducted. A total of 944 DEGs were identified, of which 457 were downregulated and 487 were upregulated in the TMPyP4 treatment group (Figure 5A).

GO analysis (Figure 5B) of the downregulated genes revealed that these genes are primarily involved in responses such as response to hormone, response to light stimulus, regulation of hormone levels, and lateral root development. They are mainly localized to the cell wall and possess functions including DNA-binding transcription factor activity, transcription regulator activity, and transmembrane transporter activity. Co-enrichment analysis of KEGG pathways (Figure 5C) showed that these genes are mainly enriched in pathways such as phenylpropanoid biosynthesis, plant hormone signal transduction, and the MAPK signaling pathway—plant. A total of 32 genes were annotated as being involved in hormone response processes, including Zm00001eb077230, Zm00001eb158740, Zm00001eb377500, Zm00001eb216860, Zm00001eb222030, Zm00001eb172450, Zm00001eb049830, Zm00001eb412510, and others.

GO analysis of the upregulated genes indicated that these genes are primarily involved in processes such as oxidation–reduction, transporter activity, secondary metabolic processes, and secondary metabolite biosynthetic processes. They are mainly localized to the plasma membrane and possess functions including oxidoreductase activity, transmembrane transporter activity and monooxygenase activity. Co-enrichment analysis of KEGG pathways revealed that these genes are predominantly enriched in pathways such as the MAPK signaling pathway—plant, phenylpropanoid biosynthesis, Diterpenoid biosynthesis and plant hormone signal transduction (Figure S2). A total of 36 genes were annotated as being involved in redox processes, including Zm00001eb385070, Zm00001eb165090, Zm00001eb176720, Zm00001eb208270, Zm00001eb140370, Zm00001eb057200, Zm00001eb409690, and others.

## 4. Discussion

The G-quadruplex ligand TMPyP4 exhibits high binding affinity for G-quadruplex structures and has been widely used to study the structure and function of G-quadruplexes [14,15,16]. TMPyP4 enhances the stability of G4 motifs by increasing their melting temperature, as demonstrated with the *c-MYC* promoter G4 or the HepB sequence of hepatitis B virus (HBV) [32,33]. TMPyP4 inhibits colorectal cancer through cell cycle arrest by targeting DG4 [15]. In this study, we applied a G-quadruplex ligand for the first time in maize to investigate G-quadruplex functions and found that TMPyP4 significantly inhibited the development of maize radicles. This indicates that TMPyP4 is involved in the mechanisms by which DG4s function in plant growth and development.

The concentrations of TMPyP4 used in this study (100, 200 and 300 µM) are higher than those typically employed in some mammalian systems, but similar doses have been widely used in microbial and plant studies. For example, Andrew et al. used 100 µM TMPyP4 in budding yeast [34], and Izbicka et al. reported that 200 µM TMPyP4 significantly reduced growth in all yeast strains tested [35]. Mikami-Terao et al. showed that 100 µM TMPyP4 strongly inhibited mammalian cell growth [36]. Considering the effects of plant cell walls and vacuoles, we used 100, 200 and 300 µM TMPyP4 in maize seedlings. The radicle growth inhibition phenotype in our study is TMPyP4-dose-dependent, showing the physiological relevance of TMPyP4 concentrations. Nevertheless, future studies should explore lower, more ecologically relevant concentrations where possible.

A major challenge in the G4 ligand field is distinguishing G4-specific effects from general cytotoxicity. TMPyP4 is known to exhibit light-dependent oxidative stress and non-specific intercalation into double-stranded DNA [34]. Our RNA-seq data showed that differentially expressed genes were significantly enriched in promoters containing known G4 motifs and in ROS-related functional categories, consistent with G4-mediated transcriptional regulation. Nonetheless, direct discrimination would require more stringent experiments, such as using G4-mutant controls, non-G4-binding analogs, or charge-reduced TMPyP4 analogs [37].

ROS can induce the formation of R-loops and promote the formation of DG4 [26]. The porphyrin TMPyP4 induces oxidative damage to G-quadruplex DNA [38]. We assessed the ROS content in radicles following TMPyP4 treatment and observed an increase in ROS levels, consistent with the findings of previous studies [31,38]. In addition, we show that TMPyP4 elevates ROS levels in radicle somatic cells, thereby inducing DNA damage in radicle cells.

A related question is whether the observed ROS changes represent a direct cause or a secondary stress response. ROS are well-known secondary signaling molecules in plant stress responses [39]. Therefore, the observed ROS elevation likely results from both direct photosensitization and secondary stress responses, which may coexist.

Bioinformatics predictions suggest that DG4s in maize regulate gene expression under various conditions, including responses to diverse stresses and DNA damage [4,40]. There are 149,988 G-quadruplex motifs predicted in the maize genome, and these motifs are predominantly located in genes associated with hypoxia response, energy signaling, oxidative stress, and inositol phosphate metabolism [4]. Volná et al. identified a highly stable G4 locus in the *RPB1* gene of Archaeplastida, conserved for over one billion years [41]. Similarly, Dobrovolná et al. showed that G4 sequences are non-randomly distributed in plant genomes and enriched in regulatory regions, suggesting functional conservation [42]. A BG4-ChIP-seq method based on the BG4 antibody has been developed and applied to identify G-quadruplex sites in rice in vitro [22]. In this study, we employed BG4-ChIP-seq to identify G-quadruplex sites in maize and found that G-quadruplex motifs are primarily enriched in genes involved in vesicle-mediated transport, regulation of jasmonic acid-mediated signaling pathways, auxin efflux, potassium ion transport, regulation of defense responses, and polar auxin transport. Integrated analysis of RNA-seq data from G-quadruplex ligand-treated radicles with BG4-ChIP-seq data revealed that at least 58% of the genes were aligned with the BG4-ChIP-seq results. Comprehensive multi-omics analysis further identified a subset of genes enriched in pathways such as energy metabolism and oxidative stress, which is largely consistent with previous data. Our data is consistent with previous studies [9,10,12] and provides critical insights into the functional roles of DG4s in maize.

Finally, regarding the agronomic and developmental relevance of the observed inhibition, G-quadruplexes play important roles in plant root development and stress responses. A study demonstrated that the Arabidopsis TOP1α protein stabilizes the G-quadruplex structure in the template strand of the CYP82C4 gene, thereby inhibiting its transcription and reducing lateral root primordium initiation. This study provides a new approach for modifying plant root system architecture [43]. Another study in grapevine (Vitis vinifera) revealed that G4s are enriched in promoters and near transcription start sites and are involved in drought stress responses [44]. Our findings provide a foundation for developing more selective and biocompatible G4-targeting compounds for crop improvement.

## 5. Conclusions

TMPyP4 inhibits maize radicle growth by increasing ROS levels and inducing DNA fragmentation. TMPyP4 modulated the expression of 1614 genes in maize radicles, which were predominantly associated with redox reactions, membrane components, and secondary metabolic pathways. DG4s are evenly distributed across the ten chromosomes of the maize genome, occupying 22,449 loci and showing significant enrichment for specific DG4-binding motifs. Integrative analysis of RNA-seq and BG4-ChIP-seq data identified 944 differentially expressed genes, which were significantly enriched in pathways related to redox reactions and secondary metabolism.

## Data Availability

All data can be made available upon request.

## Supplementary Materials

The following supporting information can be downloaded at https://www.mdpi.com/article/10.3390/life16060910/s1, Figure S1. RNA-seq sample_PCA_gene; Figure S2. GO and KEGG analysis of up-regulated genes; Figure S3. G-Score; Table S1. Primers used for RT-qPCR in the article; Table S2. Sequence alignment to the reference genome.
