# Supplementary material for: The DNA G-Quadruplex-Stabilizing Ligand TMPyP4 Inhibits Maize Radicle Growth by Modulating Reactive Oxygen Species Homeostasis
Source: Life (Basel). 2026 May 28;16(6):910. doi: 10.3390/life16060910 (PMC13301097; doi:10.3390/life16060910)
Supplement: Supplementary file 1 [file life-16-00910-s001.zip › supplementary/Figure S2. GO and KEGG analysis of up-regulated genes.pdf]

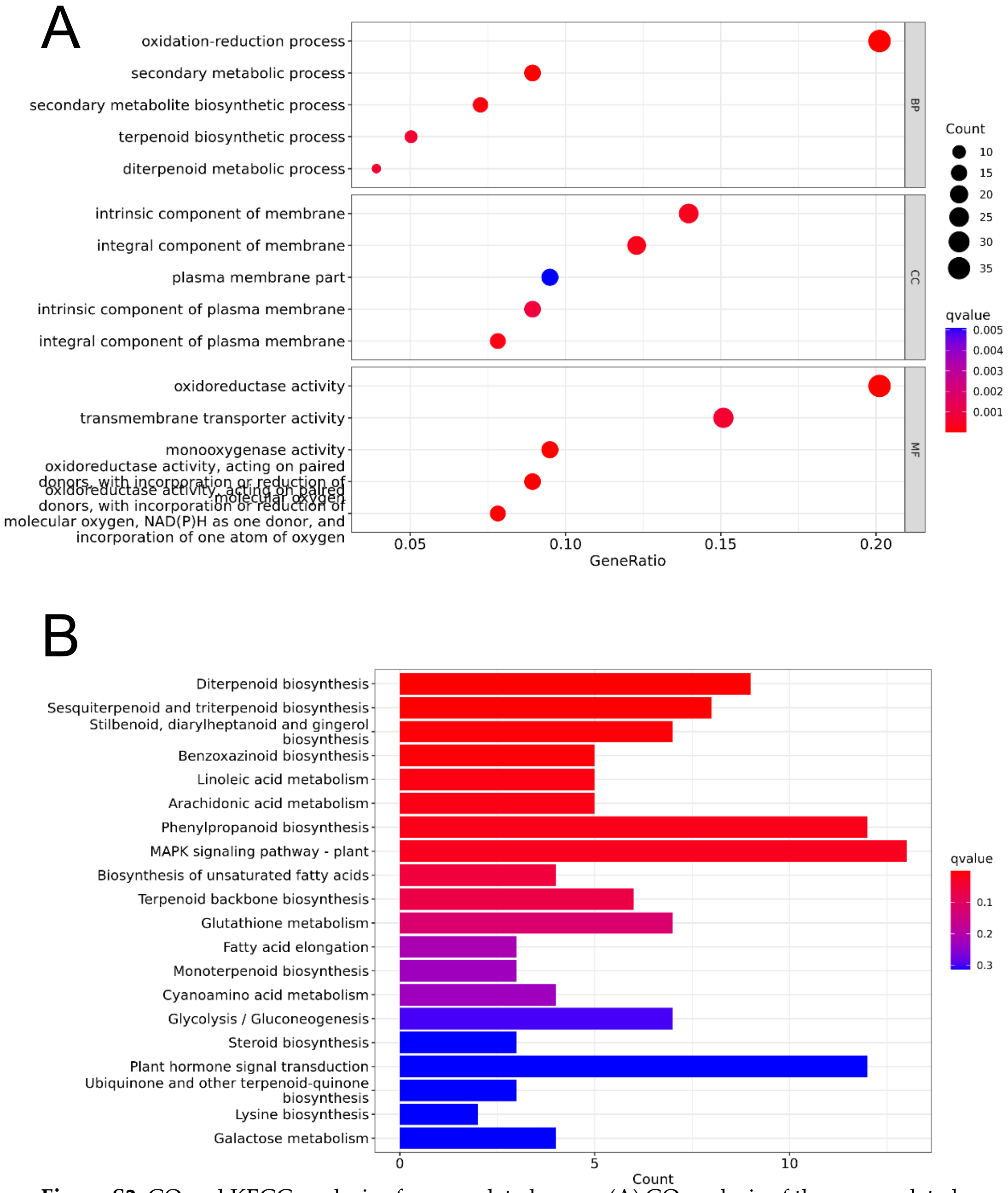


**Figure S2.** GO and KEGG analysis of up-regulated genes. (A) GO analysis of the up-regulated genes indicated that these genes are primarily involved in processes such as oxidation–reduction, transporter activity, secondary metabolic processes, and secondary metabolite biosynthetic processes. They are mainly localized to the plasma membrane and possess functions including oxidoreductase activity、transmembrane transporter activity and monooxygenase activity. (B) Co-enrichment analysis of KEGG pathways revealed that these genes are predominantly enriched in pathways such as the MAPK signaling pathway – plant 、Phenylpropanoid biosynthesis、Diterpenoid biosynthesis and plant hormone signal transduction (Appendix).
