# Supplementary material for: The DNA G-Quadruplex-Stabilizing Ligand TMPyP4 Inhibits Maize Radicle Growth by Modulating Reactive Oxygen Species Homeostasis
Source: Life (Basel). 2026 May 28;16(6):910. doi: 10.3390/life16060910 (PMC13301097; doi:10.3390/life16060910)
Supplement: Supplementary file 1 [file life-16-00910-s001.zip › supplementary/Figure S3. G-Score.pdf]

Through combined analysis of ChIP-seq and RNA-seq, genes Zm00001eb077230, Zm00001eb377500 and Zm00001eb385070 were identified, with the PEAK located in the promoter region. The promoter sequences were analyzed for G-quadruplex (G4) forming potential using G4 Hunter and QGRS Mapper, yielding a G-Score of 63, 41 and 20.

Zm00001eb077230:

Position	Length	QGRS	G-Score
15	19	GGGGCGGGGCGGGGCGGGG	63

Zm00001eb377500

Position	Length	QGRS	G-Score
25	19	GGGACGGGAGAGGGGCGGG	41

Zm00001eb385070 :

Position	Length	QGRS	G-Score
16	18	GGAGGGGAAGGGAAGAGG	20
