# Supplementary figures and images for: The DNA G-Quadruplex-Stabilizing Ligand TMPyP4 Inhibits Maize Radicle Growth by Modulating Reactive Oxygen Species Homeostasis
Source: Life (Basel). 2026 May 28;16(6):910. doi: 10.3390/life16060910 (PMC13301097; doi:10.3390/life16060910)

# PCA Plot

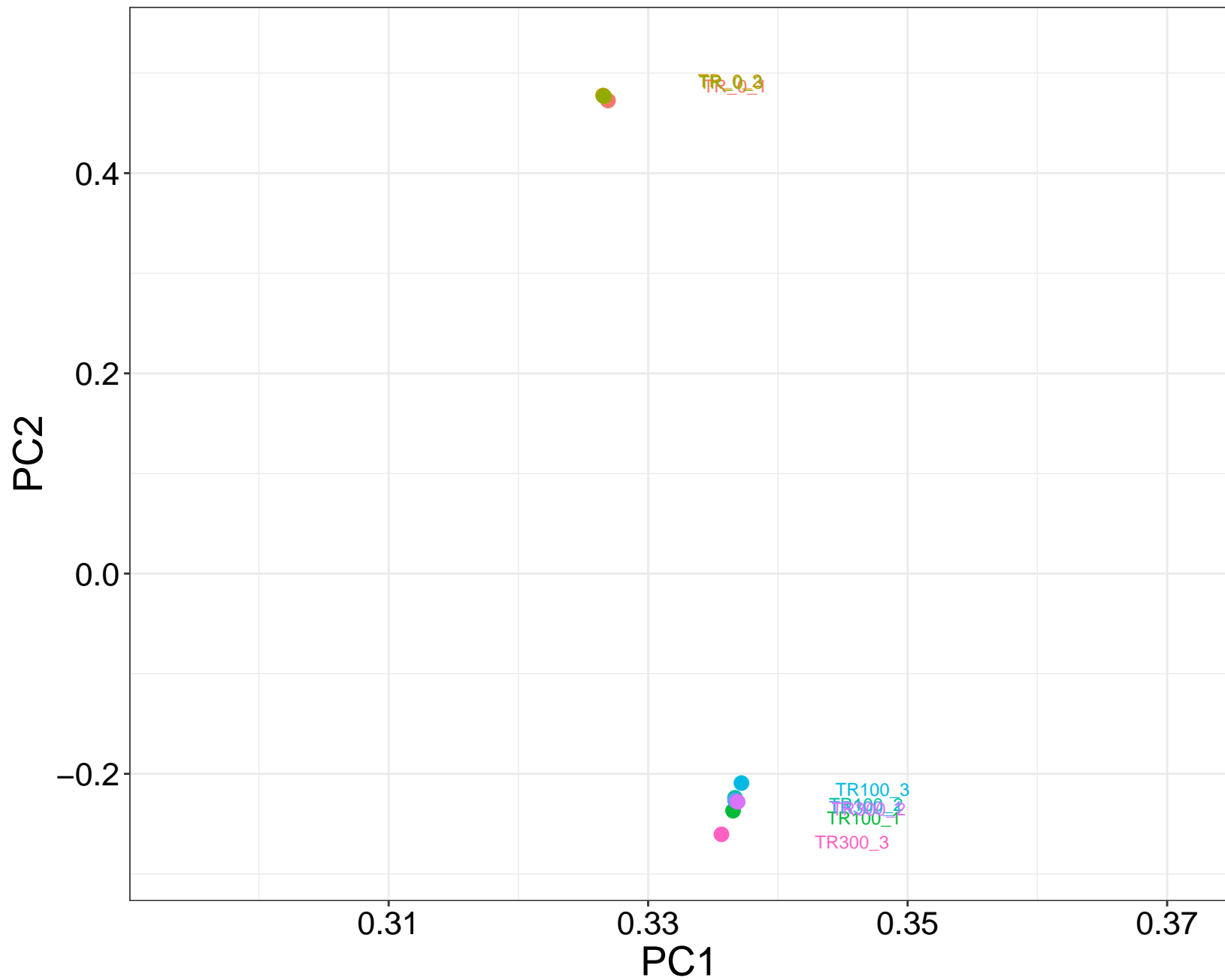

Supplement: Supplementary file 1 [file life-16-00910-s001.zip › supplementary/Figure S1. RNA-seq sample_PCA_gene.pdf]
